# Redox and pH Responsive Poly (Amidoamine) Dendrimer-Heparin Conjugates via Disulfide Linkages for Letrozole Delivery

**DOI:** 10.1155/2017/8589212

**Published:** 2017-01-26

**Authors:** Thanh Luan Nguyen, Thi Hiep Nguyen, Cuu Khoa Nguyen, Dai Hai Nguyen

**Affiliations:** ^1^Institute of Applied Materials Science, Vietnam Academy of Science and Technology, 01 Mac Dinh Chi, District 1, Ho Chi Minh City 70000, Vietnam; ^2^Can Tho University, 3/2 Street, Ninh Kieu District, Can Tho City, Vietnam; ^3^Tissue Engineering and Regenerative Medicine Group, Department of Biomedical Engineering, International University, Vietnam National University-HCMC (VNU-HCMC), Ho Chi Minh City 70000, Vietnam

## Abstract

Heparin (Hep) conjugated to poly (amidoamine) dendrimer G3.5 (P) via redox-sensitive disulfide bond (P-SS-Hep) was studied. The redox and pH dual-responsive nanocarriers were prepared by a simple method that minimized many complex steps as previous studies. The functional characterization of G3.5 coated Hep was investigated by the proton nuclear magnetic resonance spectroscopy. The size and formation were characterized by the dynamic light scattering, zeta potential, and transmission electron microscopy. P-SS-Hep was spherical in shape with average diameter about 11 nm loaded with more than 20% letrozole. This drug carrier could not only eliminate toxicity to cells and improve the drugs solubility but also increase biocompatibility of the system under reductive environment of glutathione. In particular, P-SS-Hep could enhance the effectiveness of cancer therapy after removing Hep from the surface. These results demonstrated that the P-SS-Hep conjugates could be a promising candidate as redox and pH responsive nanocarriers for cancer chemotherapy.

## 1. Introduction

The recent review of researches has been directed to the development of smart drug delivery systems in order to resolve arduous therapeutically issues associated with low treatment efficacy and high side effects [1, 2]. From 1985, Tomalia successfully synthesized the first poly (amidoamine) (PAMAM) dendrimer [3], which has been studied in recent years as multifunctional delivery system. Through stepwise synthesis process, PAMAM dendrimers (P) expressed a highly regular branching pattern and well-defined nanoscale architecture [4–6]. Furthermore, by simple chemical reactions, PAMAM dendrimers can be easily modified thanks to surface amine and ester groups forming multifunction molecules to recognize tumor cell [7, 8] and specific drug release [9, 10].

However, due to the strong interaction of the positively charged dendrimer and the negatively charged cell membrane resulting in membrane disruption, hemolytic toxicity and cell lysis are anxious disadvantages of PAMAM dendrimer drug delivery system (DDS) [1, 11]. Besides, there are also some limitations that need to be overcome (e.g., high toxicity, unsatisfied drug loading, and undesirable drug release) [12–14]. To reduce toxicity, PAMAM dendrimers were commonly modified with many different agents (PEG, heparin, fluronic, etc.) by preventing the contact between terminal protonated amine groups with cell membranes, leading to improving their biocompatibility [15–17]. By hydrophobic interactions, surface modification of heparin (Hep) is a potential way to overcome those drawbacks. Besides its typical anticoagulant activity, Hep was based on extra- and intracellular interactions to improve the bioactivity of drug and associated with protein to develop Hep-containing systems. It is also used for anticancer activities in tumor progression and metastasis processes [2, 18, 19]. Particularly, the interactions between cationic dendrimers and anionic Hep are indirectly responsible for the inhibition or enhancement of fibril formation by dendrimers. The conjugation may lead to increase in the inner cavity space of dendrimers that contributes to the increment of drug loading capacity [18, 20, 21]. In human blood circulation and cell exterior, although the disulfide bonds are generally stable, they were cleaved in a reductive environment through dithiol-disulfide exchange reactions forming thiol end-group bearing moieties [22, 23]. The cleavage reaction occurs immediately in the short time due to the high concentration of glutathione (GSH). In the cytosol, GSH is about 10 mM and higher than in extracellular nearly 500 times. Therefore, it was faster than others that elongated degrade time in the body [24–26]. The different results in various concentrations make GSH as a potential stimulus for nanodelivery system [11, 27]. In recent years, the application of the redox-sensitive mechanism on PAMAM dendrimers (G2.0 and G4.0) carrying doxorubicin and N-acetyl cysteine was found in research of Kurtoglu et al., Song et al., Hu et al., and Phuong et al. for improving a hollow core for encapsulation of drug along with biocompatibility that produced a lot of effective dendritic carrier systems [9, 28–30].

In this study, the redox-responsive DDS was designed by incorporating redox-sensitive disulfide linkage between hydrophilic Hep and PAMAM dendrimers (P-SS-Hep). The P-SS-Hep system was loaded with letrozole (Let) and could reduce systemic phenomenon and improve their focus on target at the same time. The drug system was investigated under physiological condition using proton nuclear magnetic resonance spectroscopy (^1^H NMR), dynamic light scattering (DLS), zeta potential, and transmission electron microscopy (TEM). The cleavage potential of P-SS-Hep conjugates was evaluated in different levels of GSH under reductive environment. The expected result of P-SS-Hep loaded with Let (P-SS-Hep/Let) DDS is that the nanoparticles would have the suitable size that controlled the time load and release Let. In vitro release test was also conducted to investigate effect of Hep layer on G3.5 surface.

## 2. Materials and Methods

### 2.1. Materials

Let (C_17_H_11_N_5_, 98%) and heparin sodium salt (Hep) were purchased from TCI (Tokyo, Japan). 1-Ethyl-3-(3-dimethylaminopropyl) carbodiimide (EDC), Cys dihydrochloride (C_4_H_12_N_2_S_2_·2HCl, ≥98%), L-glutathione reduced (GSH, C_10_H_17_N_3_O_6_S, ≥98%), dimethylformamide (DMF), and 4-morpholineethanesulfonic acid (MES, ≥99.5%) were purchased from Sigma-Aldrich (St. Louis, MO, USA). *α*-Bromotolunitrile, 1,2,4-triazole sodium, 4-fluorobenzonitrile, and tetra-n-butylammonium bromide (TBAB) were purchased from Merck (Darmstadt, Germany). PAMAM dendrimers generation 3.5 [G3.5(COOH)_64_] was supplied by Institute of Applied Materials Science (IAMS, Vietnam). All chemicals and solvents were of highest analytical grade and used without further purification.

### 2.2. Synthesis of Let

**Figure 1:**
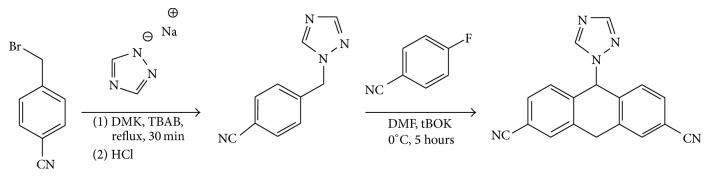
Synthetic route of Let.

Let was synthesized by utilizing *α*-Bromotolunitrile as described with some modifications (Figure 1). Briefly, *α*-Bromotolunitrile (500 mg, 2.55 mmol) was dissolved in acetone (50 mL) and then 1,2,4-triazole sodium (464 mg, 5.01 mol) and TBAB (10 mol%) were added to the solution under constant stirring. After that, the product was extracted with ethyl acetate and water to remove excess reagent. A solution of hydrochloric acid in isopropanol (1 : 1 v/v) was added dropwise to crystallize the crude product.

Thereafter, crude product (400 mg, 93% purity, and 1.69 mmol) was dissolved in DMF (20 mL). Next, potassium ter-butoxide (2.2 eq, 3.71 mmol, and 415 mg) in DMF (5 mL) and 4-fluorobenzonitrile (1.1 eq, 1.86 mmol, and 225 mg) were added under constant stirring for 5 h. Finally, the mixture was then extracted with ethyl acetate and dried to acquire a dark brown oil residue. The residue was purified using column chromatography with ethyl acetate: hexane 10–15% to acquire Let.

### 2.3. Synthesis of the P-SS-Hep

#### 2.3.1. Preparation of P-SS

Cystamine (Cys) was conjugated to PAMAM G3.5 by using EDC as coupling reagents. First, 2 g G3.5(COOH)_64_ was dissolved in 30 mL deionized water (deH_2_O), followed by adding dropwise 600 *μ*L EDC for 15 min. Secondly, the mixture was slowly added to 10 mL Cys solution (0.5 g, 3.3 mmol) at room temperature under constant stirring for 24 h. The solution was then dialyzed using a dialysis membrane (MWCO 6–8 kDa, Spectrum Laboratories, Inc., USA) against deH_2_O for 4 days at room temperature. The deH_2_O was changed 5-6 times a day and the resulting solution was then lyophilized to obtain P-SS.

#### 2.3.2. Preparation of P-SS-Hep

**Figure 2:**
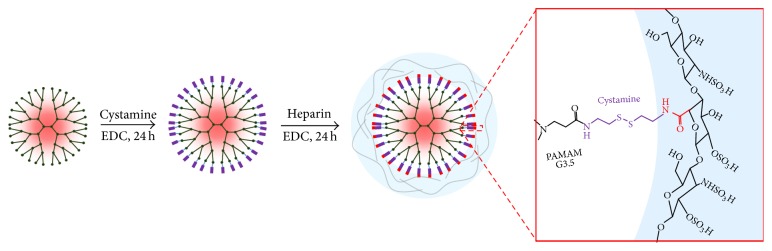
Synthetic route of the P-SS-Hep.

P-SS-Hep was synthesized under the same condition as previously described in the preparation of P-SS (Figure 2). Initially, Hep solution containing 0.8 g Hep and 10 mL deH_2_O was prepared under constant stirring. Next, 117 *μ*L EDC was immediately added to Hep solution and the reaction was carried out for 15 min. The activated Hep solution was then added to 20 mL P-SS-Hep solution (1 g, 0.07 mmol) at room temperature under stirring for 24 h. Lastly, this solution was purified by a dialysis membrane (MWCO 6–8 kDa) against deH_2_O at room temperature for 4 days. The deH_2_O was changed 5-6 times per day and the solution was freeze-dried to collect P-SS-Hep.

### 2.4. Characterization of P-SS-Hep and P-SS-Hep/Let Complexes

The chemical structure of P-SS-Hep corresponding to the synthetic procedure was analyzed by ^1^H NMR (Bruker Advance 500, Bruker Co., USA). For the purpose of investigating the presence of Cys and Hep on the surface of PAMAM G3.5, FTIR analysis (Nicolet Nexus 5700 FT-IR, Thermo Electron Corporation, Waltham, MA, USA) of PAMAM G3.5 and P-SS-Hep was carried out with KBr pellets in 400–4000 cm^−1^ range. Sizes and morphologies of PAMAM G3.5 and P-SS-Hep were assessed by Rigaku D/Max-2550 V diffractometer with Cu, K*α* radiation (*λ* = 0.15405 nm, 40 kV, and 40 mA) in the 2*θ* range from 30° to 70°. The diameters and zeta potentials of PAMAM G3.5 and P-SS-Hep were measured by a DLS (Horiba SZ-100 analyzer, Horiba Scientific Ltd., Kyoto, Japan). All samples were dissolved in 10 mM PBS (pH 7.4, 1 mg/mL), sonicated for 15 min, and measured at 37°C.

The stability of P-SS-Hep was also monitored by DLS under reductive environment. 20 mg P-SS-Hep was dissolved in 20 mL PBS (pH 7.4), followed by adding 10 mM GSH at 37°C under constant stirring. Each sample was withdrawn at predetermined time intervals (0, 15, 30, 45, and 60 min) for DLS measurement. The same procedure was repeated with 10 *μ*m GSH to examine the effect of different concentrations of GSH on P-SS-Hep.

### 2.5. Drug Loading and In Vitro Release Evaluation

The model drug Let was used in drug loading and released study according to the equilibrium dialysis method [31]. Typically, 400 mg P-SS-Hep dendrimer was immersed in 10 molar times of Let solution for 24 h under constant stirring. Then, the mixed solution was dialyzed by deH_2_O for removing unentrapped drug. The product was obtained as P-SS-Hep/Let and the drug concentration was quantified by ICP and HPLC. The entrapment efficiency (EE) and drug loading (DL) were calculated by the following equations:

(1) EE%=weight of Let in conjugatesweight of Let fed initially×100%,DL%=weight of Let in conjugatesweight of Let fed initially + weight of conjugates fed initially×100%.

In vitro release of Let from P-SS-Hep/Let was performed under four different conditions of buffers: (1) GSH 10 mM and pH 4.5, (2) GSH 10 *μ*M and pH 4.5, (3) GSH 10 mM and pH 7.4, and (4) GSH 10 *μ*M and pH 7.4. Briefly, 200 *μ*g P-SS-Hep/Let was dissolved into 10 mL deH_2_O and then dialyzed with cellulose dialysis membrane (MWCO 12–14 kDa) in couple of conditions. At certain time interval, 10 mL of each sample was taken for analysis to determine Let release by HPLC [9]. The similar concentration of free Let and dendritic solution without drug loading were dialyzed in the same condition to serve as control.

## 3. Results and Discussion

### 3.1. Characterization of Let

**Figure 3:**
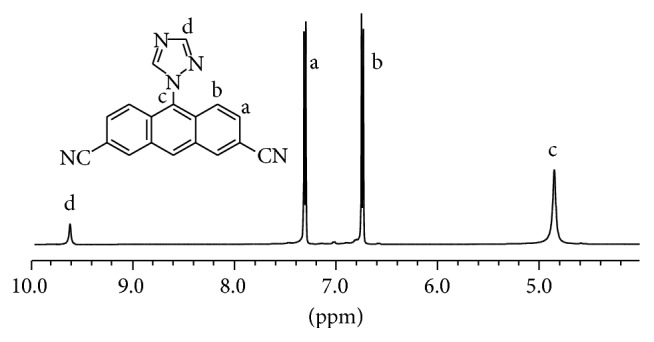
^1^H NMR spectrum of Let.

With high accuracy and impurities, the forming of Let was analyzed by ^1^H NMR that showed four distinguished singlets in Figure 3. The typical peak at 4.86 ppm H(c) was assigned to the presence of -CH_2_-C_2_N_3_H_2_- group. In addition, the appearance of the peaks at 9.61 ppm H(d) showed the tertiary proton of Let. Importantly, two strong peaks that appeared at 6.74 ppm H(b) and 7.31 H(a) ppm were attributed to the proofing effect of aromatic protons from -C_6_H_4_-CH_2_- group. The presence of all these resonance signals confirmed that the Let was successfully prepared as the pure product with high purity.

### 3.2. Characterization of P-SS-Hep

**Figure 4:**
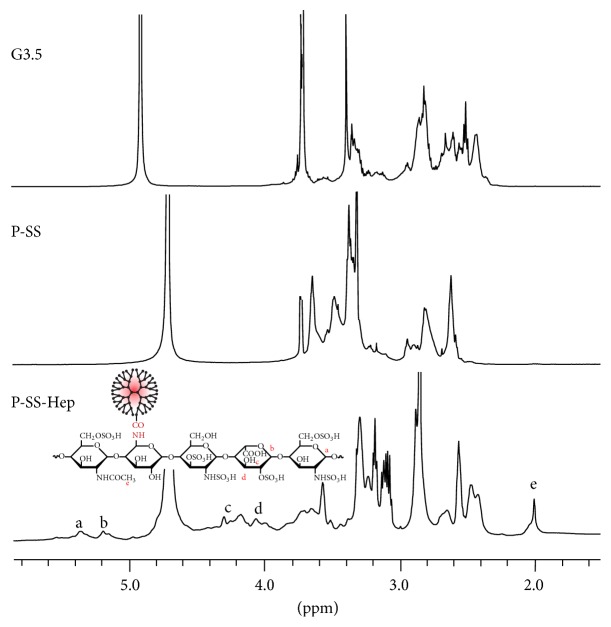
^1^H NMR spectra of PAMAM 3.5, P-SS, and P-SS-Hep.

The physicochemical properties of P-SS-Hep conjugates were characterized by ^1^H NMR, TEM, DLS, and zeta potential. As shown in Figure 4, the existence of chemical bonds between P-SS and Hep was performed by ^1^H NMR.

The peaks assigned for PAMAM G3.5 at *δ*H = 2.393–2.419 ppm, 2.475–2.501 ppm, 2.570–2.634 ppm, 2.780–2.846 ppm, 3.268–3.369 ppm, and 3.631–3.689 ppm were detectable. In comparison with P-SS spectrum, the significant change of O-CH_3_ at 3.71 ppm referred to the successful hydrolysis process of this group out of compound. Additionally, two new signals that appeared at 3.630 ppm and 3.440–3.467 ppm were attributable to the linkage between CH_2_ and S in Cys.

In ^1^H NMR spectrum of P-SS-Hep, besides the presence of typical peaks of PAMAM 3.5 and Cys, there were two proton hemiacetal signals at 5.353 ppm and 5.183 ppm. The signals at 3.988–4.290 ppm, 3.562–3.694 ppm, and 3.081–3.504 ppm were assigned to proton in hexane ring. The presence of Hep in the compound was attributed at the distinctive peak at 1.998 ppm. The presence of all these resonance signals demonstrated that P-SS-Hep was successfully synthesized.

**Figure 5:**
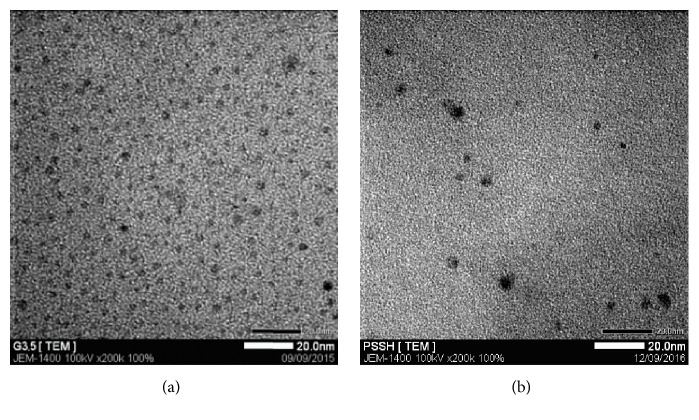
TEM images of G3.5 (a) and P-SS-Hep (b).

In addition, the morphologies and pore channel structures of the prepared particles were determined by TEM, as shown in Figure 5. G3.5 was well dispersed and P-SS nanoparticles were generally spherical-shaped in the size of range from 10 to 11 nm supporting the absorption across biological barriers, improving the capability of passive targeting through the enhanced permeability and retention (EPR) effect, and avoiding toxicity to healthy tissues and cells. This size was also consistent with DLS result and Hu et al.'s research [29] about PAMAM G4.0-PEG. The size of conjugates was around 16.9 ± 0.8 nm due to the larger size of G4.0 compared with G3.5 and longer chain of PEG compared with Hep in this study.

The nanoparticles with diameter of more than 10 nm could be formed due to crosslinking formation inside P-SS-Hep. There is a significant size increment of P-SS-Hep as compared with the PAMAM dendrimer G3.5 (about 5 nm). According to previous reports, the nanoparticles are ranging from 8 nm to 20 nm in size preferred to accumulate in effect surroundings caused by EPR effect. Furthermore, this size could prolong circulation half-life of drug because of avoidance of the recognition of stimulation immune system. These phenomena could interpret that polymers were successfully conjugated to the surface of P-SS.

### 3.3. Stability of P-SS-Hep under Reductive Environment

The redox sensitivity of P-SS-Hep was confirmed by the particle size and zeta potential under different GSH concentrations. As shown in Table 1, the zeta potential of G3.5 after being hydrolyzed with carboxylate surface groups was −15.5 mV while the zeta potential after being modified with Cys obtained positive results was +10.4 mV due to the formation of amino chloride. Neither large negative result like G3.5 nor positive value as Cys, P-SS-Hep has zeta result approximately −3.5 mV. This is explained that although most of carboxylate groups were reacted with Cys, the existence of SO_3_^−^ groups of Hep still has the ability to maintain the negative value reducing the risk of toxicity to cells.

**Figure 6:**
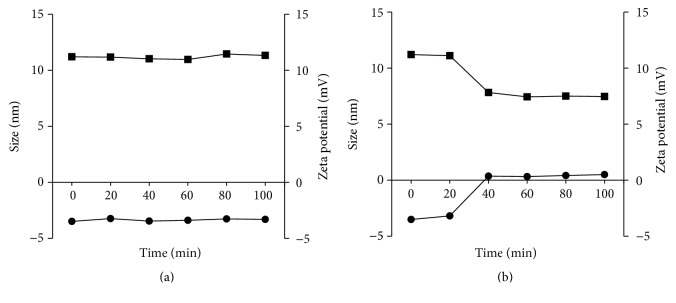
The particle sizes (square) and zeta potential changes (circle) of G3.5 and P-SS-Hep at 10 mM (a) and 10 *μ*M (b).

The differences in concentration of GSH between the intracellular tissue and plasma lead to the selective objects during drug releasing process. The effect of GSH on the cleavage of Hep is shown in Figure 6. In condition of 10 mM GSH, there is a significant reduction in size, from 11 nm to 7 nm after 40 minutes. This is because the S-S linkage was cut and the surface group of Hep was released in this condition. Meanwhile zeta potential increased from −3.51 mV to +0.5 mV that explained the S-S linkages were cleaved in 10 mM GSH. These results clearly demonstrated that, in the condition of 10 mM GSH, P-SS-Hep has high ability to break down S-S linkage in reductive environment that showed the distinct selection of P-SS-Hep during intracellular drug release.

### 3.4. Drug Loading Efficiency and In Vitro Release Profiles

The entrapment efficiency determining the drug delivery of Let and P-SS-Hep was modified. The entrapment efficiency and drug loading capacity of Let in P-SS-Hep were reached 26.21% and 6.15%, respectively. These results showed the reasonable efficiency that carriers can protect inside of drugs and prevented leaking out of the Let when they were introduced in P-SS-Hep.

**Figure 7:**
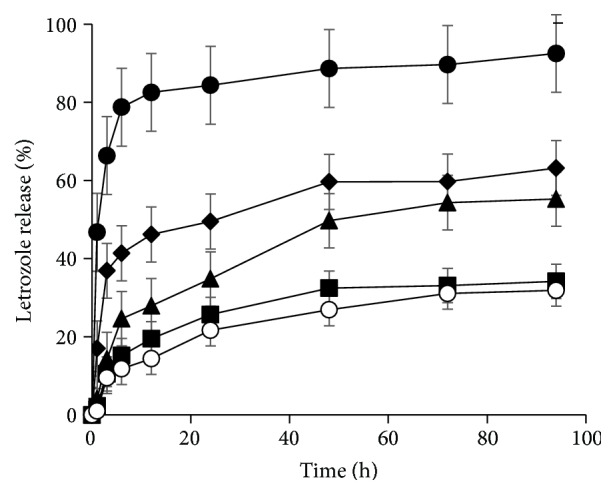
The in vitro release profiles of free Let (cycle), Let from P-SS-Hep/Let in pH 4.5 containing 10 mM GSH (lozenge), in pH 4.5 containing 10 *μ*M GSH (triangle), in pH 7.4 containing 10 mM GSH (square), and in pH 7.4 containing 10 *μ*M GSH (white cycle), respectively.

As shown in Figure 7, the release profile of the encapsulated drug molecules was investigated at 37°C under different conditions of redox and pH. Free Let was totally released during 3 h only. In the presence of 10 *μ*M and 10 mM GSH, the release of Let from the nanoparticle was remarkably different. In the condition pH 7.4, the release efficiency was quite low and the deflection was not clear in both 10 mM and 10 *μ*M. Differently, the deviation of release efficiency between 10 mM and 10 *μ*M was nearly 12% in the pH 4.5 condition due to the influence of GSH on S-S linkage.

The pH 4.5 showed the high potential to control drug release rate to cancer cells that took a longer time for Let to diffuse through the nanoparticles into the aqueous medium. At high concentration of GSH and pH, the outer Hep was detached from the conjugates upon the reductive cleavage of disulfide bonds, eliminating the steric hindrance of Let diffusion from the core to the release medium. The disulfide linkers are significantly important to improve selectivity of delivery system.

## 4. Conclusion

The redox and pH responsive P-SS-Hep conjugates containing disulfide linkers were successfully prepared and investigated by the simple and effective method. Herein, the disulfide bond has the high potential in not only reinforcing the durability during blood circulation but also enabling redox-sensitive release in the intracellular region. In cancer cells, the disulfide linkage could be effectively cleaved by antioxidant agents, resulting in the entrapped guest molecules releasing. The size of P-SS-Hep that could be controlled around 11 nm was indeed a higher effective drug loading carrier and significant potential drug release ability. Besides, the presence of Hep improves solubility and ability to pass through the membrane of the delivery system. The preliminary results show the potential of pH and redox-responsive nanocarriers in chemotherapy.

## Figures and Tables

**Table 1:** DLS and zeta potential results of G3.5, P-SS, and P-SS-Hep.

Samples	Size (nm)	Zeta potential (mV)
G3.5	5.7 ± 0.223	−15.5 ± 0.758
P-SS	7.8 ± 0.436	+10.4 ± 0.281
P-SS-Hep	10.7 ± 0.499	−3.5 ± 0.104
